# Stereotactic Ablative Radiotherapy for the Treatment of Clinically Localized Renal Cell Carcinoma

**DOI:** 10.1155/2015/547143

**Published:** 2015-11-11

**Authors:** Scott P. Campbell, Daniel Y. Song, Phillip M. Pierorazio, Mohamad E. Allaf, Michael A. Gorin

**Affiliations:** ^1^The James Buchanan Brady Urological Institute and Department of Urology, Johns Hopkins University School of Medicine, Baltimore, MD 21287, USA; ^2^Department of Radiation Oncology and Molecular Radiation Sciences, Johns Hopkins University School of Medicine, Baltimore, MD 21287, USA

## Abstract

Thermal ablation is currently the most studied treatment option for medically inoperable patients with clinically localized renal cell carcinoma (RCC). Recent evidence suggests that stereotactic ablative radiotherapy (SABR) may offer an effective noninvasive alternative for these patients. In this review, we explore the current literature on SABR for the primary treatment of RCC and make recommendations for future studies so that an accurate comparison between SABR and other ablative therapies may be conducted.

## 1. Introduction

Each year approximately 65,000 patients are diagnosed with renal cell carcinoma (RCC) in the United States [1]. Of these cases, an estimated 75% will be clinically localized at the time of presentation [2]. According to current guidelines, surgery with either partial or radical nephrectomy is the mainstay of treatment for RCC confined to the kidney [3–5]. These guidelines, however, also support the use of thermal ablation in select patients. The two major types of thermal ablation include cryoablation and radiofrequency ablation. Although less invasive than surgery, these treatment options have lower rates of local control and are therefore reserved for patients at a high competing risk of death due to advanced age or other medical comorbidities. Recently, a new ablative technique known as stereotactic ablative radiotherapy (SABR) has shown promise for the treatment of clinically localized RCC. In this review, we summarize the emerging role of SABR for the primary treatment of renal tumors. Additionally, we make recommendations for future studies so that an accurate comparison between SABR and other ablative therapies may be conducted.

## 2. Background on SABR

SABR, also known as stereotactic body radiotherapy (SBRT), is a form of hypofractionated radiation, delivering large amounts of radiation over a single or few fractions. In order to achieve the high degree of precision required to safely deliver the high doses prescribed with SABR, a variety of techniques have been utilized including custom immobilization devices with stereotactic reference systems, placement of fiducial markers for stereoscopic X-ray positioning, and/or linear accelerators equipped with on-board cone-beam image guidance. Also, methods for minimizing the effects of respiratory motion include abdominal compression systems and/or 4D computed tomography (CT) treatment simulation (which involves capturing images at various phases of the respiratory cycle). In a typical dosing scheme, 24 to 40 gray (Gy) is delivered over 1–5 fractions. This is accomplished with the patient awake and without the need for anesthesia. When only one fraction is used, the technique is referred to as stereotactic radiosurgery (SRS) [6]. SABR is delivered with a variety of modalities, most commonly with either an isocentric linear accelerator or a nonisocentric X-ray unit mounted on a robotic arm (e.g., CyberKnife, Accuray, Sunnyvale, CA). SABR or SRS is well established in the treatment of a variety of malignancies including brain tumors [7] and medically inoperable stage I non-small cell lung cancer [8].

## 3. Radiation and RCC

Despite early animal data suggesting a potential role for conventionally fractionated radiation therapy in the treatment of RCC [9, 10], clinical experience in the pre- and postnephrectomy setting has yielded mixed results (Reviewed by De Meerleer et al. [11]). Additionally, the use of standard external beam radiation therapy delivered to the kidney has been associated with unacceptably highs rate of renal and bowel toxicity [11]. Thus, at the current time guidelines do not endorse the use of radiation therapy in the management of localized RCC [3–5]. However, more recent preclinical data utilizing hypofractionated radiation [12, 13] as well as favorable experience with SABR in patients with metastatic RCC [14] have prompted a renewed interest in the use of radiation for the primary treatment of localized renal tumors.

Differences in the efficacy of conventionally fractionated radiation therapy and SABR in the treatment of RCC are likely related to their vastly different mechanisms of action. More specifically, conventionally fractioned radiation causes DNA damage that gradually leads to apoptosis. Because RCCs frequently lack the molecular control mechanisms necessary for inducing apoptosis in response to radiation-induced DNA damage [15], these tumors are relatively unaffected by conventional radiation. In contrast, SABR leads to cell death following physical damage to the cell and subsequent induction of ceramide mediated apoptotic signaling pathways [16]. Thus, while the molecular derangements of RCC may allow it to circumvent apoptosis caused by DNA damage, it is less likely to avoid the direct physical damage caused by SABR.

## 4. SABR for the Primary Treatment of Localized RCC

In total, 14 studies have been carried out to assess the efficacy of SABR for the treatment of localized RCC. These studies are summarized in Table 1. To date, the largest study which prospectively assessed the efficacy of SABR for the treatment of renal tumors included 30 cases of biopsy-proven RCC [17]. Radiation was administered in a single dose of 25 Gy with CyberKnife. Of the 30 RCC lesions treated, 6 (20%) were complete responses, 5 (17%) were partial responses, 12 (40%) were minor responses, and tumor size remained stable in 7 (23%). Estimated glomerular filtration rate (eGFR) changed minimally from a mean of 76.8 to 70.3 mL/min/1.73 m^2^ and no toxicities higher than grade I were reported.

Another noteworthy prospective study assessed SABR in 15 patients with stage I RCC [18]. This study was performed as a phase I dose-escalation study, with dosing schedules ranging from 7 Gy × 3 fractions to 16 Gy × 3 fractions. At 1 year after treatment, the breakdown of complete responses, partial responses, stable disease, and progressive disease were 1 (7%), 2 (14%), 11 (73%), and 1 (7%), respectively. With an overall median follow-up of 36.7 months, 2 local failures were reported. At 24 months, mean eGFR decreased from 55 mL/min/1.73 m^2^ to 37 mL/min/1.73 m^2^ (*p* < 0.002). No toxicities higher than grade I were reported and the two treatment failures were noted to be in the lower dose arms. Based on the lack of dose-limiting toxicities in this study, future studies with higher doses may lead to improved outcomes with little or no added morbidity.

Notably, complete responses make up only a modest percentage of lesions reported in the available literature. It is possible, however, that additional observation of noncomplete responders is required in order to appreciate the full effects of treatment. For example, one study carried out by Nomiya et al. [19] illustrated that some lesions treated with SABR continue to display modest reductions in size even as long as 40–60 months after treatment. Furthermore, while many tumors in the study exhibited growth during the first 15 months after treatment, after long-term observation every treated lesion showed an overall reduction in size. This study illustrates the importance of long-term follow-up, especially with SABR. Currently, there is little to no understanding of the long-term (>4 years) effects of SABR on RCC outside of the evidence provided by the aforementioned study.

Overall, the current literature suggests that SABR has the potential to be an effective primary treatment for clinically localized RCC. There are, however, a number of limitations to the current body of evidence worthy of mention. First, the number of patients treated is relatively small and follow-up times have generally been short. Second, outcomes have been inconsistently reported and have not been in concordance with AUA guidelines for ablative therapies for local renal masses (see below for further discussion) [20]. Third, renal function has not been consistently reported; therefore it remains unclear how well SABR spares the normal renal parenchyma. Fourth, metastases-free survival has not been consistently reported. Lastly, RCC is a heterogeneous disease with many different histologic types (e.g., clear cell RCC, papillary RCC, and chromophobe RCC), each with its own unique prognosis; thus more consistent reporting of tumor histology is required in future studies.

## 5. SABR as a Potential Alternative to Thermal Ablation

Presently, the mainstay of treatment for localized RCC is surgery with consistently reported local control rates of ≥95% with median follow-up times of five or more years [3–5]. For medically inoperable patients with small tumors, thermal ablation is an alternative option [3–5]. Compared to surgery, thermal ablation offers generally lower local control rates ranging from 83 to 95% [4, 21].

Naturally, SABR has the potential to be a less invasive equivalent to thermal ablation. To judge equivalency of these treatment modalities, however, data on SABR must be reported in similar manner to that of thermal ablation. According to a recently published guideline statement from the American Urological Association, a treatment failure is defined for ablative techniques as a “visually enlarging neoplasm or new nodularity in the same area of treatment whether determined by enhancement of the neoplasm on post-treatment contrast imaging, or failure of regression in size of the treated lesion over time, new satellite or port site soft tissue nodules, or biopsy proven recurrence” [20]. Therefore, according to these guidelines, stable disease should be reported as a treatment failure, not as a treatment success, as it is often considered in the literature on SABR. This distinction is critical when considering the efficacy of stereotactic radiation for RCC due to the slow rate of growth of untreated tumors. More specifically, small renal tumors (i.e., those ≤4 cm) have been reported to grow at a rate of only 2.5 mm per year on average [22–24]. Considering these data and the potential for error in CT measurements, ablation failures are more likely to be observed as stable lesions than progressive disease. Thus, future studies assessing SABR for primary RCC should consider using the criteria for treatment failure outlined above or alternatively utilize postablation biopsy as a measure to better judge tumor kill.

When assessing the clinical utility of a novel treatment approach, considerations beyond short-term safety and efficacy are necessary. In the case of SABR, one must also consider the salvageability of treatment failures and if the use of SABR places patients at a high risk of morbidity should surgical resection or thermal ablation be required for treatment failures. Given the relatively small number of patients treated to date, only limited data are available on this topic. Early reports do, however, suggest that surgical rescue of SABR is safe [25]. Furthermore, additional rounds of SABR have been documented to gain control of progressive lesions [18, 26].

## 6. Potential for Improvements in SABR

Data on procedural techniques of SABR for the treatment of localized RCC are limited; however, there is evidence to suggest certain methods of administration may be advantageous. For example, elevated dosing schemes are likely beneficial, as it has been consistently reported that lower doses of radiation have yielded higher rates of progression and recurrence [18, 26, 27]. Given the minimal toxicities seen in the studies to date, dosing schemes such as 40 Gy over 5 fractions are preferred. In fact, in the available literature, the highest total dose reported has been 64–80 Gy delivered over 16 fractions. Notably, in this study, all treated lesions had durable responses [19]. Future studies are, though, required to further assess the safety and efficacy of higher dosing.

In addition to improved dosing schemes, ongoing work aims to improve the accuracy of lesion targeting. A recent study by Pham et al. [28] assessed a variety of treatment plans in 20 patients receiving SABR for localized RCC. Treatment plans utilized anywhere from 8 to 13 coplanar angles and 2–9 noncoplanar angles. In addition, the authors varied the planning target volumes (PTVs) as required by the different tumor sizes. Intermediate dose fall-off (R50%, i.e., the area surrounding the PTV that receives at least 50% of the total dose) was found to be inversely correlated with both the number of beams and PTV. Thus, utilization of more beams could yield higher precision allowing for higher total doses to be administered while maintaining the same limited toxicities.

In total, vast improvements in technology and treatment planning of SABR have been made over the past decade, and continued enhancement will be necessary for SABR to be effective in the treatment of localized RCC.

## 7. Stereotactic Radiation, the Immune System, and Immunotherapy

While focal in nature, radiation has also been shown to have widespread immunologic effects. These effects have been observed both locally and in distant, nonirradiated sites [29]. When a site is irradiated and a distant tumor is extinguished this process has been deemed the “abscopal effect.” Other methods to enhance the immune system have been of great interest lately for RCC. The immune checkpoint blockade inhibitors nivolumab (anti-PD-1) and ipilimumab (anti-CTLA-4) both prevent the inactivation of cytotoxic T-lymphocytes, thus augmenting the immune response against the tumor. Early evidence suggests that the immune stimulating effects of SABR along with checkpoint blockade inhibitors may have synergistic action, and large-scale studies testing this are underway [29]. This research suggests that SABR may play a role in the curative treatment of systemic disease as an immune stimulatory agent in the future and that its effects are not limited to localized disease.

## 8. Conclusions

Radiation was once considered not only futile but also harmful in the treatment of RCC. The low doses of conventional radiation schedules did not inflict damage to tumor cells, and the lack of precision of the radiation led to significant renal and bowel toxicities. In contrast, SABR allows for the delivery of high enough doses of radiation to affect tumor survival with the precision that spares surrounding tissues. SABR is widely used for the treatment of metastatic RCC and has potential in the primary treatment of localized disease. Additional work in the way of large prospective studies is needed to better define the role of SABR in the management of patients with clinically localized RCC.

## Figures and Tables

**Table 1:** Studies of stereotactic radiotherapy as the primary treatment of localized RCC.

Study	Year	Prospective/retrospective	Dosing	Number of patients (number of lesions)	Median or mean tumor size (volume or diameter)	Median or mean follow-up (months)	Disease control^3^ (CR if reported)	Renal function	Adverse events ≥ grade 3
Qian et al. [30]	2003	Retrospective	8 Gy × 5	20 (27)	376 cc	12	93%	NR	NR

Beitler et al. [31]	2004	Retrospective	8 Gy × 5	9 (11)	4.96 cm	26.7	67%	NR	None

Wersäll et al. [32]^1^	2005	Retrospective	8 Gy × 4 10 Gy × 4 15 Gy × 3	8	NR	37	88%	Unchanged	Unclear

Gilson et al. [33]	2006	Retrospective	8 Gy × 5	14 (33)	356 cc	17	94%	NR	NR

Svedman et al. [26]^1^	2006	Prospective (phase II)	8 Gy × 4 10 Gy × 4 15 Gy × 2 15 Gy × 3	5	NR	52	60%	NR	Unclear

Ponsky et al. [27]^2^	2007	Prospective	4 Gy × 4	3	2.03 cm	NA	NA	Unchanged	None

Teh et al. [34]^1^	2007	Retrospective	24–40 Gy over 3–6 fractions	2	NR	9	100% (0%)	Unchanged	None

Svedman et al. [35]^1^	2008	Retrospective	10 Gy × 3 10 Gy × 4	7	6.04 cm	39	86%	Decreased function in 29%	None

Nomiya et al. [19]	2008	Retrospective	4-5 GyE × 16	10	4.3 cm	57.5	100% (10%)	Decreased function in 20%	1 (10%) grade 4 skin toxicity

McBride et al. [18]	2013	Prospective (phase I)	7 Gy × 3 9 Gy × 3 11 Gy × 3 13 Gy × 3 16 Gy × 3	15	3.4 cm	36.7	87% (7%)	Mean decline of 18 mg/dL	1 (6.7%) late grade 3 renal dysfunction

Nair et al. [36]	2013	Unknown	13 Gy × 3	3	21.3 cc	13	100%	Unchanged	None

Lo et al. [37]	2014	Retrospective	8 Gy × 5	3	4.77 cm	21.6	100% (0%)	Decreased function in 33%	None

Wang et al.^1^ [38]	2014	Retrospective	36–51 Gy over 10–17 fractions	9	4 cm	38.2	33% (0%)	Unchanged	None

Staehler et al. [17]	2015	Prospective	25 Gy × 1	30	33.7 cc	28.1	100% (20%)	Unchanged	None

CR, complete response; Gy, gray; GyE, gray equivalents; NA, not applicable; NR, not reported.

^1^Some or all of patients treated had metastatic lesions as well, but primary sites were treated and local control was reported.

^2^Tumors were resected after radiation; therefore eventual outcomes were not reported.

^3^Reported as the percentage of complete response, partial response, or stable disease. New metastases were counted as treatment failures. These rates cannot be directly compared to those reported for thermal ablation, which do not include stable disease as a success when calculating local control.
